# Surgical Derotation Technique: A Novel Approach in the Management of Rotated Immature Permanent Incisor

**DOI:** 10.5005/jp-journals-10005-1317

**Published:** 2015-09-11

**Authors:** Brahmananda Dutta, V Krishnapriya, CH Sriram, Maheshwar KR Reddy

**Affiliations:** Professor, Department of Pedodontics and Preventive Dentistry, Kalinga Institute of Dental Sciences, Bhubaneswar, Odisha, India; Professor, Department of Pedodontics and Preventive Dentistry, Army College of Dental Sciences, Secunderabad, Telangana, India; Reader, Department of Pedodontics of Preventive Dentistry, MNR Dental College, Hyderabad, Andhra Pradesh, India; Senior Lecturer, Department of Pedodontics of Preventive Dentistry, MNR Dental College, Hyderabad, Andhra Pradesh, India

**Keywords:** Open apex, Relapse, Splinting, Surgical derotation.

## Abstract

Surgical derotation is a method of placing a rotated tooth in normal alignment in a dental arch; surgically, immediately and permanently. It is a potentially convenient and cost-effective treatment modality as compared to conventional orthodontic procedure for rotated maxillary incisor with open apex. Here is a presentation of a severely rotated maxillary left permanent central incisor in a nine and half years old girl, with a radiographic evidence of immature root apex which was surgically derotated, orthodontically retroclined and intruded to its normal position. Postsurgical clinical and radiographic evaluation was done for a period of one and half years to confirm the vitality and continued physiological root formation of the affected tooth.

**How to cite this article:** Dutta B, Krishnapriya V, Sriram CH, Reddy MKR. Surgical Derotation Technique: A Novel Approach in the Management of Rotated Immature Permanent Incisor. Int J Clin Pediatr Dent 2015;8(3):220-223.

## INTRODUCTION

Correction of rotated incisor by orthodontic maneuver is still the most accepted technique followed today.^1,2^ Relapse, subsequent to orthodontic derotation has been well documented.^3,4^ Methods suggested to alleviate the occurrence of rotational relapse include—over correction of rotated tooth, long-term retention with bonded retainers and circumferential supracrestal fiberotomy.^3,4^ However, as per the clinical reports, none of these techniques are completely successful in preventing rotational relapse.^5-7^ In cases where teeth are severely rotated, a complex derotation mechanics as well as high level of patient compliance is also required.^8,9^ In addition to these, the major concerns for both patients and dentists are the duration of treatment for orthodontic derotation and postponement of the procedure till complete eruption of adjacent permanent teeth, so that teeth can be bonded and aligned. Alternatively, teeth can also be derotated and aligned immediately, following a minor surgical procedure. This is known as surgical derotation technique, where a tooth is luxated using forceps by giving rotational movements and derotated to its desired position, in order to achieve immediate improvement in facial esthetics with no chances of relapse. However, this technique has its own limitations and drawbacks. Firstly, teeth having conical roots, (mostly maxillary incisors and mandibular premolars) are considered ideal for such technique, as these teeth will fit into the socket uniformly following derotation without any socket modification required. Secondly, teeth with completely formed apex if derotated surgically are more likely to become non-vital as compared to open apices, where pulp vitality is maintained.^10^ This article presents a case where maxillary central incisor was surgically derotated, orthodontically retroclined and intruded. The case was then followed up for a period of one and a half years with clinical and radiographic success.

### Case Presentation

**Figs 1A and B:**
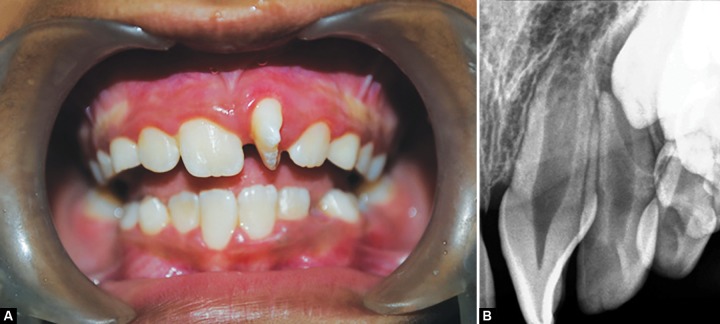
(A) Clinical picture depicting maxillary left central incisor (21) rotated mesiopalatally and (B) intraoral periapical digital radiograph showing absence of any supernumerary tooth and incomplete development of root of both 11 and 21

A nine and a half years old girl reported to the department of pedodontic and preventive dentistry (Kalinga institute of dental sciences, Bhubaneswar, India), with the chief complaint of unesthetic look of her face while smiling, due to improperly erupted upper front tooth. On clinical examination, patient was in mixed dentition stage with all 1st permanent molars and permanent incisors erupted. Maxillary left central incisor (21) was mesiopalatally rotated by 90° (Fig. 1A). Intraoral periapical digital radiograph revealed absence of any supernumerary tooth and incomplete development of root of both 11 and 21 (Nolla’s stage 8) (Fig. 1B). There was no history of trauma to the primary dentition.

### Procedure

**Figs 2A and B:**
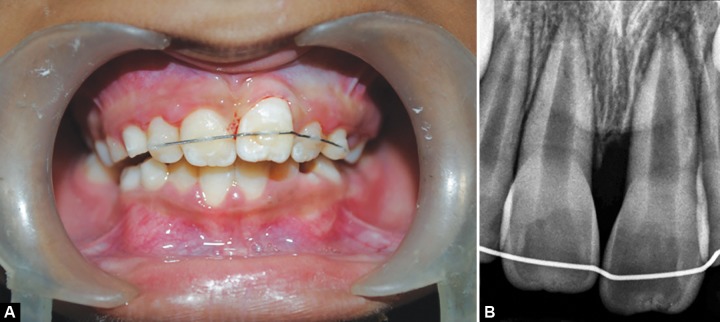
Immediate postoperative clinical picture and intraoral periapical digital radiograph showing splint

**Fig. 3:**
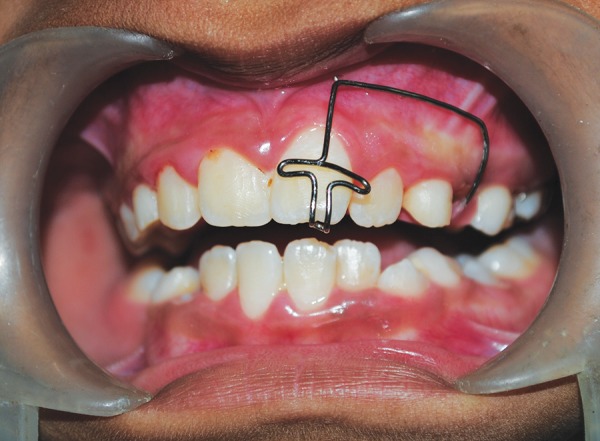
Orthodontic removable appliance (modified self supporting spring)

**Fig. 4:**
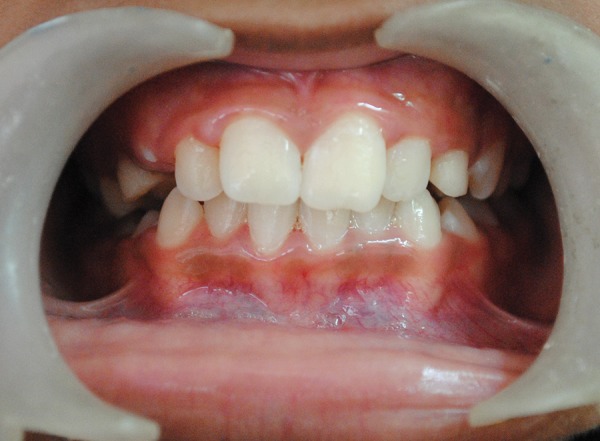
Clinical picture at 6 months follow-up

**Figs 5A to D:**
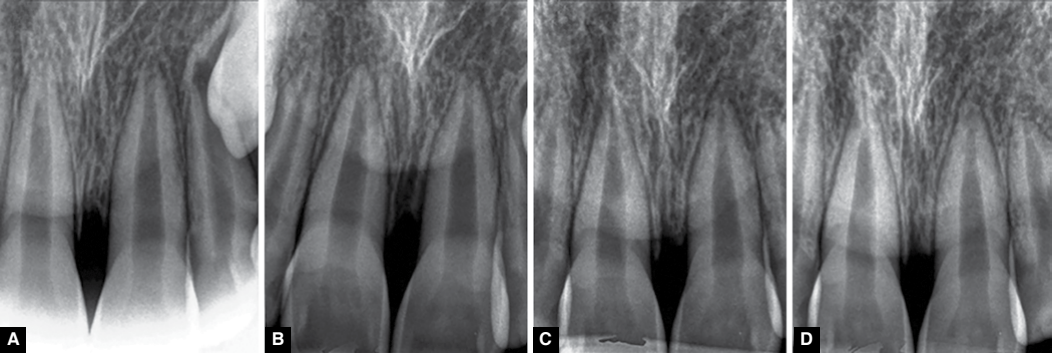
Intraoral periapical digital radiograph (A): At 3 months (B): At 6 months (C): At 12 months (D): At 18 months

Based on clinical and radiographic observations, and considering the girls concern for immediate esthetic improvement, decision was made to carry out surgical derotation technique for 21. Treatment procedure, their advantages, disadvantages and the probable post-treatment sequelae was explained to the parents and a written informed consent was taken. Procedure was carried out under local infiltration anesthesia. A root forceps was engaged on mesial and distal surfaces of the rotated tooth; it was luxated by giving rotational movements (clockwise and anticlockwise) without applying any extrusional force. Once the tooth was luxated, it was derotated clockwise to its desired position, pressing it firmly into the socket and was splinted to adjacent teeth using 0.014" NiTi wire and acid-etch technique (Figs 2A and B). Patient was discharged with a prescription of mild analgesics, chlorhexidine mouthwash and oral hygiene instructions. Splint was removed after 3 weeks and no abnormal mobility was noticed. Three months later, an orthodontic removable appliance (modified self-supporting spring) was inserted to retrocline and intrude the derotated tooth (Fig. 3). Orthodontic correction was achieved within 3 months and patient was asked to wear the same appliance for an additional 3 months as a retainer (Fig. 4). Patient was then recalled at 9, 12, and 18 months postsurgically for clinical evaluation. Radiographic evaluation was done at 3, 6, 12 and 18 months (Figs 5A to D).

### Follow-up Observations

Derotated tooth (21) was clinically evaluated for its change in color, mobility, percussion sound and pulp vitality tests (thermal and electric). All clinical parameters recorded in all the follow-up periods were normal, except vitality test, which was negative till 6 months post-derotation. However, tooth started responding to both the vitality tests from 9 months follow-up onwards.

Radiograph taken at 3 months post-derotation revealed deposition of bone with well defined lamina-dura in the apical 3rd of the developing root. An appreciable amount of root end closure with increase in root length was also observed. There was no noticeable difference in the radiographic findings between 3 and 6 months radiographs. At 12 months, pulp space in both 11 and 21 appeared to have reduced in size, indicating deposition of secondary dentin along the pulpal wall. The physiologic root growth of 21 was delayed as compared to 11. At the end of 18 months, 21 revealed radiographic root end closure.

## DISCUSSION

Surgical derotation technique, unlike orthodontic correction, has not been established as a traditional treatment technique for the management of rotated maxillary incisors because of insufficient literature. In one such case, a rotated maxillary incisor which was intruded following a trauma, was surgically repositioned and simultaneously derotated.^11^ However, post-treatment follow-up was not reported.

The present technique has several advantages over orthodontic derotation like—simple, cost-effective and stable; besides, there is immediate esthetic improvement. Once the tooth is derotated surgically, root of the involved tooth is detached from all the periodontal and gingival fibers, including epithelial attachment. Within 2 to 3 weeks, periodontal and gingival fibers reorganize themselves and new attachments are formed at the corrected position of the tooth, which is evidenced by normal percussion sound.^12^ Blood vessels and nerve fibers entering through the apical foramen also get severed and may not regenerate again if apex is completely formed, leading to pulp necrosis. However, tooth with open apex revascularization and regeneration of nerve fibers can be predicted.^13,14^ Revascularization can be confirmed radiographically by evidence of continued root formation and pulp canal obliteration. Similarly, a return to a positive pulp response to sensitive testing is a clinical sign of nerve regeneration.

Before carrying out surgical derotation of a tooth, factors need to be assessed are: degree of rotation of the affected tooth, developmental stage of the root and space availability for its alignment. In the case presented here, tooth was rotated by 90° and root-end closure was yet to be completed. Space available was sufficient to accommodate the M-D width of the crown. Therefore, this case was suitable for surgical de-rotation technique to be undertaken. A thin (0.014") semi-rigid wire was used for splinting to allow physiologic tooth movement, which in turn, enhance healing process and prevents ankylosis.^15^ A 3-month observation period was followed prior to the inception of orthodontic tooth movement, as recommended by the authors.^16,17^ Periodic periapical radiographs revealed continued root-end closure of the derotated tooth, but at a slower pace as compared to adjacent contralateral tooth. Probable explanation for this delay would be an initial localized transient disturbance of cells around the root apex, as a result of trauma following derotation, and thereafter due to pressure exerted from orthodontic appliance. As all the clinical parameters and radiographic findings were positive both at 12 and 18 months, the present surgical technique was considered as successful.

## CONCLUSION

Success of surgical derotation technique as presented in this article clearly emphasizes that this technique certainly warrants more consideration. However, more studies with long-term follow-up are required for this method to be recommended. Also, prognosis of this technique in mature teeth needs to be experimented.
